# Maternal Vitamin D Status and Its Effect on Vitamin D Levels in Early Infancy in a Tertiary Care Centre in Sri Lanka

**DOI:** 10.1155/2019/9017951

**Published:** 2019-07-09

**Authors:** Kaneshapillai Anusha, Usha Hettiaratchi, Dulani Gunasekera, Shamini Prathapan, Guwani Liyanage

**Affiliations:** ^1^Department of Biochemistry, Faculty of Medical Sciences, University of Sri Jayewardenepura, Sri Lanka; ^2^Department of Paediatrics, Faculty of Medical Sciences, University of Sri Jayewardenepura, Sri Lanka; ^3^Department of Community Medicine, Faculty of Medical Sciences, University of Sri Jayewardenepura, Sri Lanka

## Abstract

Epidemiologic studies from South Asian countries have reported vitamin D deficiency among all age groups. However, there is very little information on vitamin D levels, especially in the vulnerable populations (pregnant/breast feeding mother and infants) in Sri Lanka. More data on vitamin D status of such populations will be important for policy decisions to be made at a national level. Similarly, it will be valuable for healthcare programs in other countries (e.g., United States, Australia, Europe, and Canada) as Sri Lankans are a fast-growing migrant population to those countries. The purpose of this study was to investigate maternal vitamin D status and its effects on infants in a state sector tertiary care centre in Sri Lanka. This prospective cohort study was conducted on 140 healthy pregnant mothers in the third trimester (mean gestational age 39±1 weeks). Blood was collected for 25(OH)D and parathyroid hormone (PTH). Sun exposure and feeding patterns of the infants were recorded based on maternal reporting. Mean age of the infants at follow-up visit was 36±7 days. Vitamin D (25 (OH)D) deficiency (<25 nmol/L) was observed in 12% pregnant mothers, 5% lactating mothers, and 63% infants. Insufficiency (<50 nmol/L) was found in an additional 51% and 43% in pregnant and lactating mothers and 25% of infants. Mean 25(OH)D was higher in pregnant (46.4±17.5 nmol/L) and lactating (51.9±17.0 nmol/L) mothers than infants (28.1±13.7 nmol/L). Maternal vitamin D level during pregnancy was a significant risk factor (OR: 6.00, 95%CI: 1.522-23.655) for infant deficiency and insufficiency. Sun exposure of infants showed a significant positive correlation with vitamin D level (OR: 3.23, 95%CI: 1.19-8.68). In conclusion, the presence of Vitamin D deficiency/insufficiency is higher in infants compared to pregnant/lactating mothers. Low maternal 25(OH)D during pregnancy was a risk factor for deficiency in infants. Although majority of lactating mothers had sufficient vitamin D, most of their exclusively breastfed offspring were deficient.

## 1. Introduction

Countries near the equator receive more sunlight throughout the year. However, sun-seeking behavior is uncommon in these countries due to the hot climate. Epidemiological studies from our neighboring country, India, have shown high prevalence of low vitamin D (25 OHD (< 50 nmol/L) in all age groups: neonates, preschool and school children, pregnant women, and adult males [1]. In Sri Lanka, Rodrigo et al. have reported that 56% of premenopausal women have vitamin D level ≤35 nmol/L in the Southern coastal belt of the country [2]. In recent years, vitamin D level <50 nmol/L was reported among preschoolers (35%) and pregnant mothers (63%) [3, 4].

Growing infants are more vulnerable to develop vitamin deficiencies. Cultural influences maintain a high breastfeeding rate in our country. Thus, the nutrition of infants solely depends on the maternal levels during their first 6 months of exclusive breastfeeding period. Vitamin D deficiency among infants and their breastfeeding mothers is not reported in Sri Lanka to date. Vitamin D deficiency leading to rickets and hypocalcaemic seizures has been shown among breastfed infants in Southern India [5, 6]. Atiq et al. have reported that 55% of breastfed infants had 25 OHD below 25 nmol/L in Pakistan [7].

There are no guidelines on vitamin D supplementation during pregnancy, infancy, or other age groups in Sri Lanka [2–4]. Food fortification with vitamin D is not common in the country except for a few food items such as infant formula, fat spreads, and baby cereal products. Sri Lankans are a fast-growing migrant population to Western countries (e.g., United States, Australia, Europe, and Canada). They are at a higher risk of vitamin D deficiency since they are moving to countries in higher latitudes away from the equator. It is important that healthcare organizations of these countries are aware of this community of dark skinned individuals, who are at risk of vitamin D deficiency.

Thus, this present study was undertaken to determine the vitamin D status in pregnant mothers, lactating mothers, and their infants and to investigate the effect of maternal vitamin status on infant vitamin D levels, in a tertiary care centre in Sri Lanka.

## 2. Method

### 2.1. Study Design, Setting, and Sample

Healthy women aged 18 years or more with singleton pregnancies in their 3rd trimester were recruited for this prospective cohort study at the antenatal clinic in a teaching hospital in Colombo district. Based on a previous study, sample size was calculated [8]. This teaching hospital is a state sector tertiary care centre in the Southern part of Colombo district. Clinics in state-run hospitals offer calcium lactate, iron fumarate, and folic acid only, as supplements during pregnancy. Subjects were enrolled through a convenient sampling technique. Mothers with uncomplicated pregnancies who were not on vitamin D supplementation were recruited for the study. Ethical clearance for the study was obtained from the Ethics Review Committee, Faculty of Medical Sciences, University of Sri Jayewardenepura, Sri Lanka (Reference No: 22/16). Informed written consent was obtained from all participants. Procedures performed in this study involving human participants were in accordance with the ethical standards of the institutional research committee.

Following informed written consent, a pre-tested interviewer administered questionnaire was used to collect information on socio-demography, nutrition, past and present health and well-being. A physical examination, including anthropometry, was performed on all mothers at enrollment. Blood samples (4 ml) were collected from the eligible pregnant mothers and serum was stored at -20°C for laboratory analysis.

### 2.2. Follow-Up at 4-6 Weeks of Postpartum

Infant-mother pairs were reviewed at 4-6 weeks of postpartum. Reminders via telephone calls (maximum of 3) and a stipend for transport were provided for these parents. Prematurity, congenital anomalies, or any neonatal problems in babies requiring high dependency were considered as exclusion criteria. Detailed physical examination of the infant, including weight, length, head circumference (OFC), and size of the anterior and posterior fontanel was recorded. Sun exposure and feeding (exclusive breastfeeding, formula feeding, and mixed feeding) of the infant were recorded based on maternal reporting. The approximate duration (in minutes) of sun exposure per day, frequency (days) per week, and infant clothing were taken to quantify the amount of sun exposure. Blood samples were drawn from both lactating mothers and their infants for laboratory analysis.

### 2.3. Laboratory Analysis

Biochemical analysis was performed at the University of Sri Jayewardenepura, Colombo, using the following methods:

1. Vitamin D3 (25OHD) was measured by VIDAS® 25 OH Vitamin D Total using the Enzyme Linked Fluorescent Assay (ELFA). It has good correlation with the Liquid Chromatography-Mass Spectrometry reference method with good cross reactivity with vitamin D2 (91%) and D3 (100%).
2. Intact PTH was measured by DRG (EIA-3645) ELISA method. Intra-assay variability is 6.08% for low concentration (32.4 pg/mL) and 3.68% for higher concentration (178.2 pg/mL). Interassay variability is 3.6% for low concentration (30.3 pg/mL) and 2.85 for higher concentration (159.1 pg/mL).
3. Analysis of calcium, inorganic phosphorous (IP), and alkaline phosphatase (ALP) was performed by colorimetric method using the Thermo Scientific Konelab 20XT Analyzer.

### 2.4. Data Analysis

Data were analyzed using the Statistical Package for the Social Sciences (SPSS) version 15.0. Vitamin D deficiency (VDD), vitamin D insufficiency (VDI), and sufficiency are defined as < 25 nmol/L, 25-50 nmol/L, and ≥50 nmol/L of 25OHD levels, respectively [9, 10]. PTH cut-off value was considered as 66.5pg/mL as per manufacturer's recommendations. ALP cut off was taken as 420 IU/L for infants, 100 IU/L for lactating mothers, and 250 IU/L for pregnant mothers [11, 12]. Chi square test, Spearman correlation, and independent sample t-test were used for analysis. Statistical significance was set at 95% confidence interval (*P *<0.05).

The STROBE cohort reporting guidelines were used during the manuscript preparation.

## 3. Results

We recruited a total of 140 pregnant mothers. Twenty-five (18%) mothers/infants were excluded due to following reasons: not attending the follow-up visit (n=19), prematurity (n=3), congenital anomalies (n=1), and receiving neonatal intensive care (n=2). A final sample included 115 mothers and 112 infants (consent was not given for blood sampling for 3 infants).

Mean (SD) age of the infants at follow-up visit was 36±7 days. Almost equal gender distribution (M:F of 54:58) was observed among them. Mean (SD) age of the pregnant mothers was 29 (±6) years. Most (81%) mothers were housewives. Majority of mothers were Sinhalese (77.4%) and had either primary or secondary education (93.9%). Almost all of the study population is from low and middle income families (89%).

### 3.1. Infant Data

Mean values of 25OHD, PTH, and biochemical parameters are given in Table 1. Majority (63%) of the infants had VDD (Table 2). However, secondary hyperparathyroidism was observed only in 9% of vitamin D deficient infants. Serum mineral concentrations (calcium and inorganic phosphorus) were within the normal range in all. ALP was more than 420 IU/L in 42% of infants. Significant but weak negative correlation was observed between vitamin D and PTH. However, correlation between ALP and vitamin D was not significant (Table 3).

Mean weight at birth and at follow up visit were 3000 ± 440 g and 4063 ± 713 g respectively. In Sri Lanka less than 2500 g is considered as low birth weight. Low birth weight was observed in 12.2%. The mean weight gain of the population was 1060 ± 544 g. However, two babies from this study population showed poor weight gain at follow-up visit.

Correlations between 25 OHD and weight (r = -0.172, p=0.069) and OFC (r =-1.61, p=0.089) at follow-up visit were not significant. Although correlation between length and vitamin D level was positively correlated, this finding was not statistically significant (r= 0.198, p=0.557). All these correlations were analyzed after eliminating confounding factors (gestational age, gender of the baby, and the height of the mother). None of the infants had evidence of rickets (craniotabes, wide skull sutures, rachitic rosary, and enlargement of the wrists and ankles) or hypocalcaemic convulsions.

Most of the infants (85.2%) were exposed to the sun either with (only a diaper) or without clothes. None of the babies have used sunscreen in this population. Maximum duration of exposure was 60 minutes/day. Duration of sun exposure had a significant effect on infant vitamin D levels (odds ratio: 3.23, 95% CI: 1.19-8.68). Majority (98.3%) of the infants was exclusively breastfed and two babies were on mixed feeding (formula feeding and breastfeeding).

### 3.2. Maternal Data

VDD was observed more during pregnancy than lactation (Table 2). Mean 25OHD has improved from 46.4±17.5 nmol/L in pregnancy to 51.9±17.0 nmol/L during lactation. Total alkaline phosphatase (heat labile and heat stable) was more than the cut off levels (pregnancy: 250 IU/L, lactation: 100 IU/L) in 8% and 77% during pregnancy and lactation, respectively). There was no significant difference between serum calcium and phosphate both during pregnancy and lactation. Correlation between 25OHD and ALP and PTH is shown in Table 3. PTH level was above the cut off (>66.5 pg/mL) in 4% & 19% in pregnant and lactating mothers, respectively.

### 3.3. Comparison of Maternal and Infant Data

The odds ratio was calculated to compare maternal and infant vitamin D levels. Maternal deficiency or insufficiency during pregnancy was a significant risk factor for infant vitamin D deficiency/insufficiency (OR: 6.000; 95%CI: 1.522-23.655; p value: 0.009). Lactating mother's vitamin D level was not seen as a risk factor for infant vitamin D deficiency/insufficiency (OR: 3.122; 95%CI: 0.789-12.217; p value: 0.127).

## 4. Discussion

This study reports high rates (88%) of vitamin D deficiency/insufficiency (<50 nmol/L) among infants in a single centre in Western Sri Lanka. Jain et al. have reported high rate of vitamin D deficient mothers (81%) and their infants aged 2.5-3.5 months (66.7%) in New Delhi, India [13]. Vitamin D deficiency in exclusively breastfed infants (12-26%) has been reported from Southern India [5, 14].

There are limited data on vitamin D levels in children and adults in Sri Lanka. Marasinghe et al. have reported <50 nmol/L among 35% in preschool children (2-5 years) in a study conducted in the Western Province [3]. Lower rate of deficiency in these preschoolers compared to infants in our study could be due to the increase in sun exposure and higher vitamin D content in their diet as children grow older. Further, Rodrigo et al. have reported that 56% of premenopausal women had vitamin D deficiency (cut off value of 35 nmol/L) in Southern Sri Lanka [2]. Authors had concluded that the high rate of deficiency in that population could be due to the sample being limited to women who were employed and who spend most of the day indoors. In our cohort, most women were housewives. They generally have a different pattern of sun exposure. This could be the likely reason for lower deficiency rate among pregnant/lactating mothers in our study.

25(OH)D passes from the placenta into the bloodstream of the fetus. Many studies have shown that maternal 25(OH)D during pregnancy has a good correlation with cord blood levels at birth [15–17]. In the present study, maternal vitamin D levels during pregnancy correlated with vitamin D levels in early infancy.

After birth, vitamin D status of an exclusively breast fed infant depends on vitamin D of milk and sun exposure. Human breast milk and unfortified cow's milk have very little vitamin D [18]. Vitamin D content in breast milk is <20 IU/L and this level is not adequate for the needs of the growing infant [19]. An exclusively breastfed infant will receive <20% of the daily requirement through breast milk [20]. Thus, the poor correlation of 25(OH)D observed between the infant and the lactating mother in this study could have been due to low transfer of maternal 25(OH)D through breast milk. In addition, mothers during lactation had higher mean level of 25(OH)D than during their pregnancy. Possible reason for this observation could have been due to less transfer of 25(OH)D though breast milk compared to placental transfer.

However, the supply of 25(OH)D through breast milk increases when vitamin D supplements are provided [21, 22]. A double blind prospective study in USA has reported decreased bone mineral content of infants receiving breast milk alone compared to breast milk with additional supplementation [23]. When lactating women are given 4000–6000 IU/day of vitamin D, enough vitamin D transferred in breast milk to satisfy the infant's requirement [24].

Clinical Practice Guidelines in Australia do not provide vitamin D supplementation to all exclusively breastfed newborns unless there are additional risk factors [25]. However, guidelines of many other countries recommend prenatal and postnatal vitamin D supplementation. In the United Kingdom, infants are routinely supplemented with vitamin D [26]. American Academy of Pediatrics has recommended breastfed infants to be supplemented with 400 IU/day of vitamin D in the first few days of life [27]. Clinical Practice Guidelines of Endocrine Society, United States recommend at least 600 IU/day of vitamin D during pregnancy. A minimum of 1400–1500 IU/day of vitamin D is needed for lactating mothers to satisfy the requirements of infant, if additional vitamin D supplements are provided to the infant. If the infant is not supplemented, mother requires high doses of vitamin D as supplementation (4000-6000 IU/day) to transfer adequate amounts of vitamin D to breast milk [21]. However, the higher doses of vitamin D supplementation (>10,000 IU/day) can increase the serum 25OHD drastically and can lead to hypercalcaemia [28].

Kovacs has reported that vitamin D deficiency during pregnancy and lactation can lead to hypocalcaemia and rickets in infants [29]. In this study, none of the infants had hypocalcaemia. They had higher mean level of ALP indicating mobilization of calcium from bone. However, ALP did not show a significant correlation with vitamin D level. Thus, it indicates that ALP is not reflective of serum vitamin D levels. However, ALP is frequently used as a surrogate marker of vitamin D deficiency in pediatric practice, especially when managing preterm babies, in Sri Lanka.

Expected rise of PTH was not seen in the majority of the study population. We considered the threshold for secondary hyperparathyroidism to be 66.5pg/mL. Souberbielle et al. have shown that PTH starts to rise >46 pg/mL when 25OHD is falling below 30 nmol/L [30]. However, measurement of PTH concentration in identifying the optimal level of vitamin D remains controversial. Hence, we need further research to clarify the clinical value of PTH in identifying vitamin D deficiency/insufficiency.

The American Academy of Pediatrics has recommended that infants below six months should be kept out of direct sunlight because of growing concerns about over exposure to sun and skin cancer [27]. However, the people in Sri Lanka believe that neonatal jaundice is prevented or treated by exposure to the sun. Thus, it is common that most of the parents expose their newborns to the morning sunlight. Since the exposure to the sun is the major source of vitamin D, details of infant sun exposure were recorded and there was a positive correlation between exposure time and vitamin D levels. Jain et al. have shown that sunlight exposure, with the baby wearing only a diaper, for 30 min/week can raise the serum 25OHD to ≥27.5 nmol/L [13]. In our study, infants who were exposed to sunlight >30min/week (72%) had a higher mean level of 25OHD (29.5±14.3 nmol/L) compared to those who were with reduced exposure (24.8±11.7 nmol/L).

Vitamin D deficiency/insufficiency is a likely risk factor for obesity in children [31, 32]. Diane et al. have reported that vitamin D deficiency is associated with increased weight gain in school-children [28]. A preliminary analysis of this study showed maternal vitamin D deficiency had no correlation with infant anthropometry. Furthermore, infant vitamin D status did not show a correlation with weight, length, and OFC as indicated in the results [33].

There were a few limitations in this study. Present sample did not represent all ethnic, sociodemographic, and geological variations in Sri Lanka. Study sample was restricted to mother-infant pairs from lower and middle socioeconomic backgrounds in an urban setting who seek care from state sector hospitals. Further, due to nonavailability of equipment and funds the bone mineral content, density, and nonskeletal effects such as immune dysfunction were not assessed in this cohort of pregnant mothers and their infants.

## 5. Conclusions

The present study shows that vitamin D deficiency/insufficiency is seen among pregnant and lactating mothers and their offspring from a tertiary care centre in Sri Lanka. However, in contrast to mothers, infant vitamin D deficiency was higher. Maternal vitamin D deficiency during pregnancy was a risk factor for vitamin D deficiency in infants. We suggest further studies on vitamin D levels in a nationally representative sample of mothers and infant pairs to confirm the present findings to take a step forward towards formulation of guidelines for supplementation. Moreover, investigations into the suitability of the current cut-off values for our population, long-term impact of vitamin D deficiency in infants, and biological significance of PTH as a marker of vitamin D deficiency are other important areas for future research.

## Figures and Tables

**Table 1:** Biochemical parameters of the study population.

	25(OH)D nmol/L	PTH pg/mL	IP mmol/L	ALP IU/L	Calcium mmol/L
Pregnant mothers (n=115)	46.4 ± 17.5	23.7 ± 22.0	1.3 ± 0.2	193.6 ± 172.0	2.3 ± 0.2
Lactating mothers (n=115)	51.9± 17.0	41.2 ± 38.1	1.3 ± 0.2	121.1 ± 25.4	2.2 ± 0.1
Infants (n=112)	28.1 ± 13.7	28.6 ± 22.9	2.1 ± 0.2	415.7 ± 107.6	2.5 ± 0.1

Mean±SD unless otherwise indicated.

25(OH)D: vitamin D; PTH: Parathyroid Hormone; IP: Inorganic Phosphorous; ALP: Alkaline Phosphatase.

**Table 2:** Low 25 (OH)D levels in pregnant/lactating mother and infants.

Groups	VDD (25(OH)D <25 nmol/L)	VDI (25(OH)D 25-50 nmol/L)
Pregnant mothers (n=115)	14 (12)	58 (50.9)
Lactating mothers (n=115)	06 (5)	49 (42.6)
Infants (n=112)	71 (63)	28 (25.0)

Number (%) unless otherwise indicated.

VDD: Vitamin D Deficiency; VDI: Vitamin D Insufficiency.

**Table 3:** Correlation between serum 25(OH)D and other biochemical parameters.

Group	Correlation r (p value)			
	PTH	ALP	Calcium	IP
Pregnant mothers (n=115)	- 0.295 (0.002)^*∗*^	- 0.084 (0.392)	- 0.019 (0.845)	0.163 (0.096)
Lactating mothers (n=115)	- 0.249 (0.011)^*∗*^	- 0.165 (0.092)	- 0.068 (0.492)	- 0.068 (0.490)
Infants (n=112)	- 0.283 (0.004)^*∗*^	- 0.067 (0.502)	0.122 (0.221)	0.244 (0.013)^*∗*^

^*∗*^significantly correlated.

25(OH)D: vitamin D; PTH: Parathyroid Hormone; IP: Inorganic Phosphorous; ALP: Alkaline Phosphatase.

## Data Availability

The data used to support the findings of this study are included within the article.

## References

[1] Nimitphong H., Holick M. F. (2013). Vitamin D status and sun exposure in Southeast Asia. *Dermato-Endocrinology*.

[2] Rodrigo M., Hettiarachchi M., Liyanage C., Lekamwasam S. (2013). Low serum vitamin D among community-dwelling healthy women in Sri Lanka. *Health*.

[3] Marasinghe E., Chackrewarthy S., Abeysena C., Rajindrajith S. (2015). Micronutrient status and its relationship with nutritional status in preschool children in urban Sri Lanka. *Asia Pacific Journal of Clinical Nutrition*.

[4] Anusha K., Guwani L., Hettiaratchi U., Dulanie G. (2018). Impact of diet on vitamin D status in a Sri Lanka-based sample of pregnant mothers. *J Health Soc Sci*.

[5] Balasubramanian S., Shivbalan S., Saravana Kumar P. (2006). Hypocalcemia due to vitamin D deficiency in exclusively breastfed infants. *Indian Pediatrics*.

[6] Hemalatha R., Anchoju V. C., Donugama V. (2018). Maternal vitamin D status and neonatal outcomes. *The Indian Journal of Pediatrics*.

[7] Atiq M., Suria A., Nizami S. Q., Ahmed I. (1998). Maternal vitamin-D deficiency in Pakistan. *Acta Obstetricia et Gynecologica Scandinavica*.

[8] Meyer H. E., Holvik K., Lofthus C. M., Tennakoon S. U. (2008). Vitamin D status in Sri Lankans living in Sri Lanka and Norway. *British Journal of Nutrition*.

[9] Royal-College-of-Paediatrics-&-Child-Health (2013). *Guide for Vitamin D in Childhood UK2013*.

[10] Arundel P., Shaw N. (2015). *Vitamin D and Bone Health: A Practical Clinical Guideline for Management in Children and Young People*.

[11] Tran H. A. (2005). Abnormal laboratory results, Biochemical tests in pregnancy. *Austaralian Prescriber*.

[12] Kahl L., Hughes H. K. (2017). *The Harriet Lane Handbook E-Book*.

[13] Jain V., Gupta N., Kalaivani M., Jain A., Sinha A., Agarwal R. (2011). Vitamin D deficiency in healthy breastfed term infants at 3 months & their mothers in India: Seasonal variation & determinants. *Indian Journal of Medical Research*.

[14] Tergestina M., Jose A., Sridhar S. (2014). Vitamin D status and adequacy of standard supplementation in preterm neonates from south India. *Journal of Pediatric Gastroenterology and Nutrition*.

[15] Rodda C. P., Benson J. E., Vincent A. J., Whitehead C. L., Polykov A., Vollenhoven B. (2015). Maternal Vitamin D supplementation during pregnancy prevents Vitamin D deficiency in the newborn: an open-label randomized controlled trial. *Clinical Endocrinology*.

[16] Bowyer L., Catling-Paull C., Diamond T., Homer C., Davis G., Craig M. E. (2009). Vitamin D, PTH and calcium levels in pregnant women and their neonates. *Clinical Endocrinology*.

[17] Ariyawatkul K., Lersbuasin P. (2018). Prevalence of vitamin D deficiency in cord blood of newborns and the association with maternal vitamin D status. *European Journal of Pediatrics*.

[18] Wang J., Yang F., Mao M., Liu D., Yang H., Yang S. (2010). High prevalence of vitamin D and calcium deficiency among pregnant women and their newborns in Chengdu, China. *World Journal of Pediatrics*.

[19] Kamao M., Tsugawa N., Suhara Y. (2007). Quantification of fat-soluble vitamins in human breast milk by liquid chromatography–tandem mass spectrometry. *Journal of Chromatography B*.

[20] við Streym S., Højskov C. S., Møller U. K. (2015). Vitamin D content in human breast milk: a 9-mo follow-up study. *The American Journal of Clinical Nutrition*.

[21] Holick M. F., Binkley N. C., Bischoff-Ferrari H. A. (2011). Evaluation, treatment, and prevention of vitamin D deficiency: an endocrine society clinical practice guideline. *The Journal of Clinical Endocrinology & Metabolism*.

[22] Wall C. R., Stewart A. W., Camargo C. A. (2015). Vitamin D activity of breast milk in women randomly assigned to Vitamin D3 supplementation during pregnancy. *American Journal of Clinical Nutrition*.

[23] Greer F. R., Searcy J. E., Levin R. S., Steichen J. J., Asch P. S., Tsang R. C. (1981). Bone mineral content and serum 25-hydroxyvitamin D concentration in breast-fed infants with and without supplemental vitamin D. *Journal of Pediatrics*.

[24] Hollis B. W., Wagner C. L. (2013). Vitamin D and pregnancy: skeletal effects, nonskeletal effects, and birth outcomes. *Calcified Tissue International*.

[25] The Royal Children's Hospital (2019). *Clinical Practice Guideline on vitamin D deficiency*.

[26] Spiro A., Buttriss J. L. (2014). Vitamin D: an overview of vitamin D status and intake in Europe. *Nutrition Bulletin*.

[27] Perrine C. G., Sharma A. J., Jefferds M. E., Serdula M. K., Scanlon K. S. (2010). Adherence to vitamin D recommendations among US infants. *Pediatrics*.

[28] Tebben P. J., Singh R. J., Kumar R. (2016). Vitamin D-mediated hypercalcemia: mechanisms, diagnosis, and treatment. *Endocrine Reviews*.

[29] Kovacs C. S. (2008). Vitamin D in pregnancy and lactation: Maternal, fetal, and neonatal outcomes from human and animal studies. *The American Journal of Clinical Nutrition*.

[30] Souberbielle J.-C., Lawson-Body E., Hammadi B., Sarfati E., Kahan A., Cormier C. (2003). The use in clinical practice of parathyroid hormone normative values established in vitamin D-sufficient subjects. *The Journal of Clinical Endocrinology & Metabolism*.

[31] Gilbert-Diamond D., Baylin A., Mora-Plazas M. (2010). Vitamin D deficiency and anthropometric indicators of adiposity in school-age children: a prospective study. *American Journal of Clinical Nutrition*.

[32] Al-Daghri N. M., Sabico S., Al-Saleh Y. (2016). Calculated adiposity and lipid indices in healthy Arab children as influenced by vitamin D status. *Journal of Clinical Lipidology*.

[33] Anusha K., Hettiaratchi U. P., Liyanage G., Gunasekera D. P. (2018). Vitamin D status of pregnant mothers and its effect on anthropometric measures in the offspring: a preliminary study. *Sri Lanka Journal of Child Health*.

